# Comparison of Postoperative Outcomes between Leadless and Conventional Transvenous Pacemakers Implantation: An Up-to-Date Meta-analysis

**DOI:** 10.31083/j.rcm2510359

**Published:** 2024-10-09

**Authors:** Huimiao Dai, Hao Liu, Chuncheng Gao, Jing Han, Jun Meng, Pengyun Liu, Mingming Zhang, Dongdong Li, Wangang Guo

**Affiliations:** ^1^Department of Cardiology, The Second Affiliated Hospital of Air Force Medical University, 710038 Xi’an, Shaanxi, China; ^2^Xi’an Medical College,710000 Xi’an, Shaanxi, China; ^3^Department of Cardiology, Shenzhen Hospital of Southern Medical University, 510086 Shenzhen, Guangdong, China

**Keywords:** conventional transvenous pacemakers, leadless pacemaker, meta-analysis, postoperative outcomes, systematic review

## Abstract

**Background:**

Leadless cardiac pacemakers (LCPs) are emerging as viable alternatives to conventional transvenous pacemakers (TVPs). This study aimed to systematically compare the postoperative outcomes of LCPs and TVPs based on available published studies.

**Methods:**

We conducted a systematic review and meta-analysis of literature comparing outcomes from LCP and TVP implantations. Data analysis was performed using Stata/MP 17.0. The evaluated endpoints included pericardial effusion or perforation, puncture site events, infective endocarditis, lead or device dislodgement, pocket-related complications, tricuspid regurgitation or dysfunction, any infection, increased right ventricle (RV) pacing threshold, embolism, and thrombosis. Aggregated odds ratios (OR) and 95% confidence intervals (CI) were determined. Sensitivity analyses were conducted for heterogeneity if I^2^ was >50% or *p* < 0.01, otherwise, the random-effects model was chosen. Publication bias was analyzed if the number studies exceeded ten.

**Results:**

The meta-analysis included 24 observational studies with 78,938 patients, comprising 24,191 with LCP implantation and 54,747 with TVP implantation. The results indicated a significantly lower incidence of lead or device dislodgment (OR = 3.32, 95% CI: 1.91–5.77, *p* < 0.01), infective endocarditis (OR = 3.62, 95% CI: 3.10–4.24, *p* < 0.01), and infection (OR = 3.93, 95% CI: 1.67–9.24, *p* < 0.01) in the LCP group compared to the TVP group. In contrast, incidences of puncture site complications (OR = 0.24, 95% CI: 0.19–0.32, *p <* 0.01) and pericardial effusion or perforation (OR = 0.33, 95% CI: 0.28–0.39, *p* < 0.01) were significantly higher in the LCP group.

**Conclusions:**

Compared with TVP, LCP implantation is associated with a lower risk of infective endocarditis, lead or device dislodgment, infections, and pocket-related complications. However, LCP implantation carries a higher risk of puncture site complications and pericardial effusion or perforation. These findings underscore the need for careful consideration of patient-specific factors when choosing between LCP and TVP implantation.

**The PROSPERO Registration:**

https://www.crd.york.ac.uk/prospero/ (CRD42023453145).

## 1. Introduction

Leadless cardiac pacemakers (LCPs) are emerging as an alternative to conventional transvenous pacemakers (TVPs), primarily due to their potential to reduce specific complications. Unlike TVP’s which require the creation of a subcutaneous pocket to advance the lead through the subclavian vein, LCPs eliminate the need for both, thereby avoiding risks associated with pocket-related issues such as bleeding, infection, rupture, pneumothorax, hemothorax, and lead dislodgment [1, 2]. Furthermore, TVPs are generally recommended for patients in relatively good physical condition, excluding individuals with conditions like permanent left superior vena cava, subclavian or superior vena cava thrombosis or occlusion, significant tricuspid regurgitation (TR), tricuspid valve replacement, elderly patients, and those at high risk of bleeding or infection [3, 4, 5, 6]. Although LCP implantation circumvents these pocket and lead-related complications, its use of a thicker and stiffer delivery system introduces other risks, including puncture site hematoma, arterio-venous (AV) fistula, pseudoaneurysm, and pericardial perforation [7, 8].

This meta-analysis aims to provide a comprehensive comparison of the complication profiles associated with leadless and traditional pacemakers in patients with bradyarrhythmia, highlighting their advantages and disadvantages. Such insights are crucial for enabling patients and healthcare professionals to make informed decisions regarding pacemaker selection. The most recent meta-analysis, published on April 27, 2023, involved a systematic literature search up to April 12, 2022 [9]. Since then, numerous large- scale studies have enriched the body of evidence concerning leadless pacemakers [8, 10, 11, 12, 13, 14, 15, 16, 17, 18]. This study not only assessed standard outcomes but also explored additional endpoints, including TR or dysfunction, pocket-related complications, and infection rates. Employing the latest methodological standards, our systematic review and meta-analysis was thoroughly conducted and is registered with PROSPERO (CRD42023453145) (https://www.crd.york.ac.uk/prospero/).

## 2. Methods

### 2.1 Literature Searching

A systematic literature search was performed through PubMed, Ovid Medline, Web of Science, Embase, Cochrane, China National Knowledge Infrastructure, Wanfang and Weipu from inception to August 3, 2023. The following key words were used for the search: “‘leadless pacemaker’ OR ‘leadless pacing’ OR ‘micra’ OR ‘nanostim’ AND ‘transvenous pacemaker’ OR ‘traditional pacemaker”’. Moreover, the relevant references were evaluated. The search strategy is presented in **Supplementary Table 1**.

### 2.2 Inclusion and Exclusion Criteria

Inclusion criteria included: (1) studies comparing postoperative outcomes between the two pacemaker types; (2) both randomized controlled trials (RCT) and observational studies (OSs) were considered. Exclusion criteria included: (1) studies lacking the specified observation endpoints; (2) studies where full texts were unavailable; (3) studies reporting on the same population multiple times; (4) studies with incomplete or ambiguous data; (5) studies deemed low-quality by two independent reviewers.

### 2.3 Data Extraction

The data from the studies was extracted by HD and HL, who then independently and rigorously assessed their quality. Any arising conflicts were resolved through thorough discussion with CG. The extracted data encompassed: (1) fundamental details about the enrolled studies, such as the study period, and study type; (2) general characteristics of the subjects, including the sample size, mean age, sex distribution, and follow-up period; (3) specified outcomes of interest.

### 2.4 Endpoints and Definitions

The endpoints included: incidence of complications, including pericardial effusion or perforation, puncture site events (hematoma, AV fistula, pseudoaneurysm), infective endocarditis, lead/device dislodgment, pocket-related complications (hematoma, infection, ulceration), TR or dysfunction, any infection, increased right ventricle (RV) pacing threshold, embolism and thrombosis (deep venous thrombosis [DVT]), cardiac device-related pulmonary embolism or thrombosis.

Incidents of TR or dysfunction are defined as the occurrence of new TR or an increase in severity during follow-up compared to the time of implantation. Infection is characterized by systemic and device-related infection. An increase in RV pacing threshold is defined as a rise greater than 2-fold from the value when the pacemaker was implanted. Embolism and thrombosis refer to deep venous thrombosis (DVT), cardiac device-related pulmonary embolism or thrombosis.

### 2.5 Risk Assessment of Bias

Both HD and HL conducted bias risk assessment, and CG resolved any conflict. The original plan of this study was to include both randomized controlled trials and observational studies, but after screening, only observational studies were included. Observational studies were rigorously evaluated using the Newcastle-Ottawa Quality Assessment Scale (NOS) [19].

### 2.6 Statistical Analysis

EndNote X9 (Clarivate Analytics, Philadelphia, PA, USA) was used for literature management. The aggregated odds ratios (OR) were calculated at 95% confidence intervals (CI) using STATA/MP 17.0 (StataCorp, College Station, TX, USA). The incidences of postoperative complications in the LCP and TVP groups were compared based on OR and 95% CI. Heterogeneity among the studies was assessed via the I^2^ test. Notably, the fixed-effects model was employed when *p* > 0.01 and I^2^ < 50%, otherwise, the random-effects model was chosen. The origin of heterogeneity was evaluated via heterogeneity test and sensitivity analysis. Publication bias was assessed using funnel plots and a regression-based Egger test. *p* value < 5% was deemed statistically significant.

## 3. Results

### 3.1 Literature Search Results and Baseline Information

**Fig. 1.**
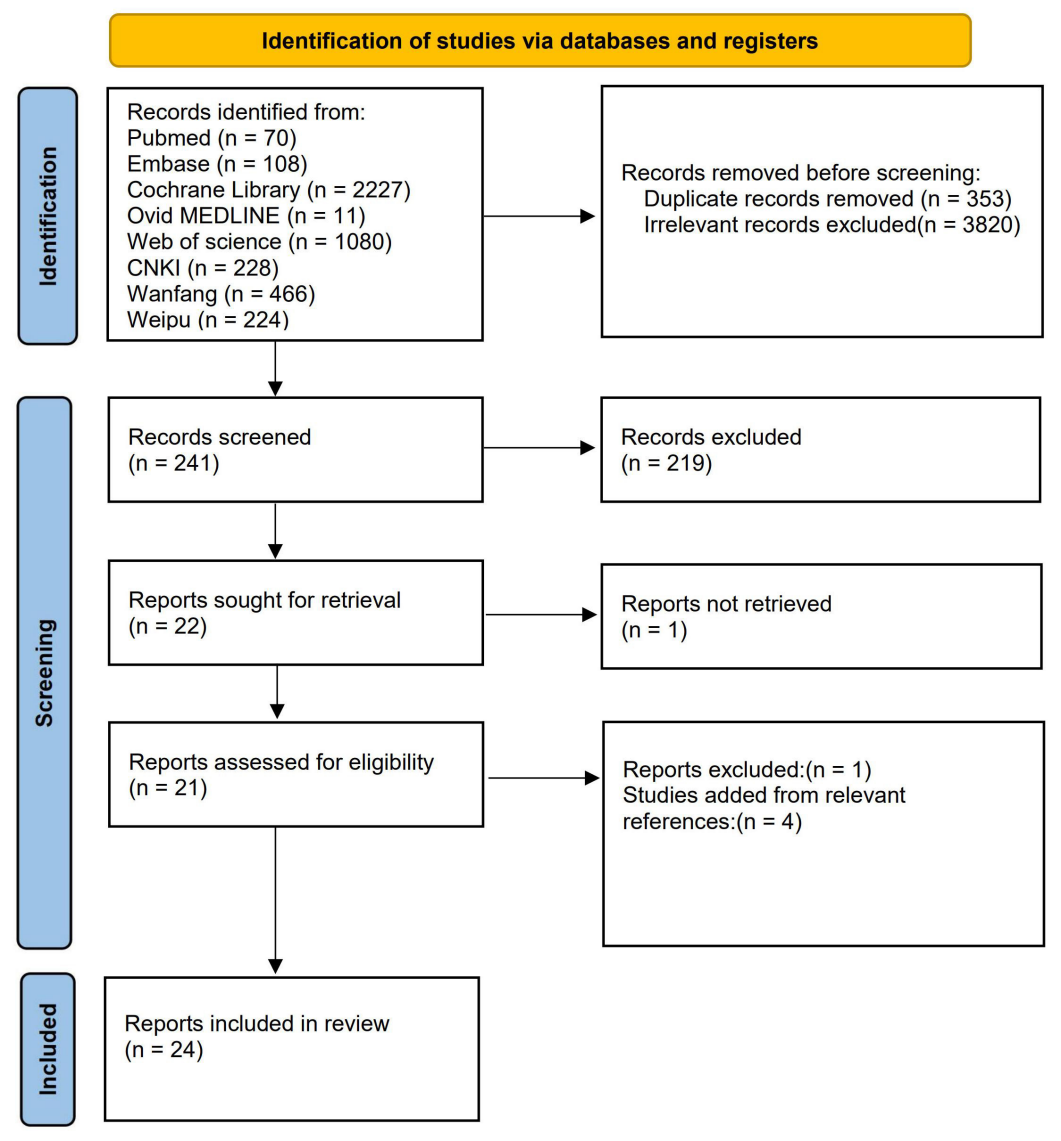
**Flowchart of literature selection process for meta-analysis**. The flowchart in Fig. 1 outlines the comprehensive process of literature selection undertaken for the meta-analysis. Initially, the Identification phase involves retrieving records from eight distinct databases summing up to a total of 4414 articles. In this stage, records that were removed prior to screening are also noted. The Screening phase follows, where 241 records were screened, leading to the exclusion of 219 records. The next step involved seeking 22 reports for retrieval, of which one could not be retrieved. This phase continues with the assessment of 21 reports for eligibility, resulting in one exclusion and the addition of four studies based on relevant references. The process culminates in the Included phase, where 24 reports are finalized for inclusion in the review. This systematic approach ensures a thorough and meticulous selection of studies pertinent to the research question addressed by the meta-analysis.

A total of 4414 studies were initially identified from eight databases. From these, 353 were excluded due to duplication, 3820 were excluded due to relevance, 219 for meeting the exclusion criteria, and one for not meeting the inclusion criteria. An additional study was excluded because its full text was unavailable and its design (not a large cohort study) limited its generalizability to our research question. However, four studies were added after reviewing relevant references. Consequently, 24 studies [8, 10, 11, 12, 13, 14, 15, 16, 17, 18, 20, 21, 22, 23, 24, 25, 26, 27, 28, 29, 30, 31, 32, 33] were ultimately included in our analysis. These comprised 19 published in English and four in Chinese. The process of study selection is detailed in Fig. 1. The collective baseline information for the participants in these studies is shown in Table 1 [8, 10, 11, 12, 13, 14, 15, 16, 17, 18, 20, 21, 22, 23, 24, 25, 26, 27, 28, 29, 30, 31, 32, 33]. All included studies were observational, and published between 2016 and 2023.

**Table 1.** **Baseline characteristics of the enrolled studies and subjects**.

Study	Study type	Study period	Country/Territory	Sample size	Male, %	Mean age, years	Follow-up period
				(LCP/TVP)	(LCP/TVP)	(LCP/TVP)	
Piccini *et al*. [8], 2021	retrospective cohort	Mar.2017–Jun.2018	USA	5746/9662	56.3%/56.6%	79.4 (9.5)/82.0 (8.1)	6 months
Lu *et al*. [10], 2022	retrospective cohort	Jan.2020–Sep.2020	China	6/18	66.7%/61.1%	76.8 ± 10.5/73.5 ± 11.8	1 month
Yang *et al*. [11], 2022	retrospective cohort	Sep.2019–Apr.2021	China	36/72	52.8%/54.2%	68.6 ± 13.4/70.7 ± 11.2	3 months
Liang *et al*. [12], 2021	retrospective cohort	Aug.2018–Dec.2018	China	15/31	53.3%/58.1%	68.5 ± 11.9/68.2 ± 9.8	12 months
Chen *et al*. [13], 2022	prospective cohort	Dec.2019–Dec.2020	China	60/10	62%/60%	68.92 ± 13.14/66.82 ± 14.32	4 months
Turagam *et al*. [14], 2020	retrospective cohort	Jan.2014–Oct.2018	USA	24/48	8.4%/10.5%	32.7 ± 6.2/31.6 ± 5.3	3, 6, 12 months
Zucchelli *et al*. [20], 2021	retrospective cohort	May.2014–Apr.2019	Italy	100/100	77%/67%	77.46 ± 9.58/78.78 ± 9.78	1, 6, 12 months
Bodin *et al*. [15], 2022	retrospective cohort	Jan.2017–Sep.2020	France	1344/1344	58.6%/59.7%	73.5 ± 17.2/73.5 ± 15.2	6.2 ± 8.7 months
Vaidya *et al*. [21], 2019	retrospective cohort	2014–2017	USA	90/90	63%/63%	80.5 (74.0–86.0)/78.2 (73.8–85.3)	62/180 days
Pagan *et al*. [22], 2020	retrospective cohort	Dec.2015–Nov.2019	USA	183/119	51.9%/40.3%	89.5 ± 3.4/89.9 ± 3.4	1 week
Tachibana *et al*. [23], 2020	cohort	May.2014–Jul.2019	Japan	27/35	44.4%/40.0%	90.1 ± 3.5/90.9 ± 4.1	6 months
Cantillon *et al*. [24], 2018	retrospective cohort	Feb.2014–Jan.2016	USA	718/1436	62.3%/63%	75.9 ± 11.9/76.1 ± 12.3	323/408 days
		Apr.2010–Mar.2014					
Tjong *et al*. [16], 2018	retrospective cohort/prospective	Dec.2012–Dec.2016	the Netherlands	220/220	60.9%/60.5%	78.0 (70.0–84.0)/77 (69–82)	599/1486 days
Moore *et al*. [25], 2019	retrospective cohort	Feb.2017–Aug.2018/Jul.2008–Aug.2018	USA	10/23	60%/52%	82.8 (3.1)/84 (1.4)	84 ± 32/199 ± 87 days
Beurskens *et al*. [17], 2019	retrospective cohort	Jan.2013–May.2018	the Netherlands	53/53	70%/70%	80.5 ± 7.92/79.3 ± 6.89	365 ± 30 days
Sanchez *et al*. [26], 2021	retrospective cohort	Feb.2014–Jun.2019	USA	67/131	54%/27%	73 ± 16/74 ± 10	817 ± 600/592 ± 549 days
El-Chami *et al*. [27], 2022	retrospective cohort	Mar.2017–Dec.2018	USA	6219/10,212	55.9%/56.8%	79.5 ± 9.5/82.0 ± 8.1	2 years
Sasaki *et al*. [28], 2022	retrospective cohort	Sep.2017–Sep.2020	Japan	58/58	38%/40%	81 ± 8/82 ± 6	801/649 days
Yarlagadda *et al*. [29], 2018	prospective cohort	Feb.2014–Nov.2016	USA	60/67	48%/24%	74 ± 8.7/74 ± 9.6	12 months
Martinez-Sande *et al*. [30], 2021	prospective cohort	Jun.2015–Dec.2019	Spain	198/245	62.1%/27.3%	79.2/83.6	22.3 ± 15.9 months
Palmisano *et al*. [31], 2021	prospective cohort	May.2016–Dec. 2019	Italy	442/442	68.3%/68.8%	73.2 ± 15.3/74.0 ± 12.6	39 months
Reynolds *et al*. [32], 2016	prospective cohort	May.2015	International	725/2667	58.8%/55.1%	75.9 ± 10.9/71.1 ± 12.1	4 months
Okuyama *et al*. [33], 2020	retrospective cohort	Jan.2016–Mar.2019	Japan	10/14	20%/21.4%	86.5 (83.5–90.3)/83 (81–87)	6 months
Alhuarrat *et al*. [18], 2023	cohort	2016–2019	USA	7780/27,650	57%/56%	77.1 ± 12.1/81.3 ± 9.4	2 years

LCP, leadless cardiac pacemaker; TVP, transvenous pacemaker.

### 3.2 Quality Assessment

The quality of the observational studies was assessed, using the Newcastle-Ottawa Scale (NOS), as shown in Table 2 [8, 10, 11, 12, 13, 14, 15, 16, 17, 18, 20, 21, 22, 23, 24, 25, 26, 27, 28, 29, 30, 31, 32, 33]. A higher score indicates a higher quality of research. Quality scores are categorized as follows: 0–3 indicates low quality, 4–6 indicates medium quality, and 7–9 indicates high quality. The studies included in this analysis consistently demonstrated a low risk of bias.

**Table 2.** **Quality assessment of the cohort studies using NOS**.

Study	Selection	Comparability	Outcome	Total score
Piccini *et al*. [8], 2021	★★★★	★★	★★	8
Lu *et al*. [10], 2022	★★★★	★	★★	7
Yang *et al*. [11], 2022	★★★★	★	★★	7
Liang *et al*. [12], 2021	★★★★	★	★★★	8
Chen *et al*. [13], 2022	★★★★	★	★★	7
Turagam *et al*. [14], 2020	★★★	★	★★★	7
Zucchelli *et al*. [20], 2021	★★★	★★	★★★	8
Bodin *et al*. [15], 2022	★★★★	★★	★★	8
Vaidya *et al*. [21], 2019	★★★★	★★	★★	8
Pagan *et al*. [22], 2020	★★★	★	★★	6
Tachibana *et al*. [23], 2020	★★★	★	★★	6
Cantillon *et al*. [24], 2018	★★★★	★	★★	7
Tjong *et al*. [16], 2018	★★★★	★★	★★	8
Moore *et al*. [25], 2019	★★★	★	★★	6
Beurskens *et al*. [17], 2019	★★★★	★	★★★	8
Sanchez *et al*. [26], 2021	★★★	★	★★★	7
El-Chami *et al*. [27], 2022	★★★★	★★	★★★	9
Sasaki *et al*. [28], 2022	★★★	★★	★★★	8
Yarlagadda *et al*. [29], 2018	★★★	★	★★★	7
Martinez-Sande *et al*. [30], 2021	★★★★	★★	★★	8
Palmisano *et al*. [31], 2021	★★★★	★★	★★★	9
Reynolds *et al*. [32], 2016	★★★	★	★★	6
Okuyama *et al*. [33], 2020	★★★★	★	★★	7
Alhuarrat *et al*. [18], 2023	★★★★	★★	★★	8

NOS, Newcastle-Ottawa Quality Assessment Scale; ★, when a certain condition is met, one star is awarded.

### 3.3 Primary Endpoints

#### 3.3.1 Puncture Site Events

**Fig. 2.**
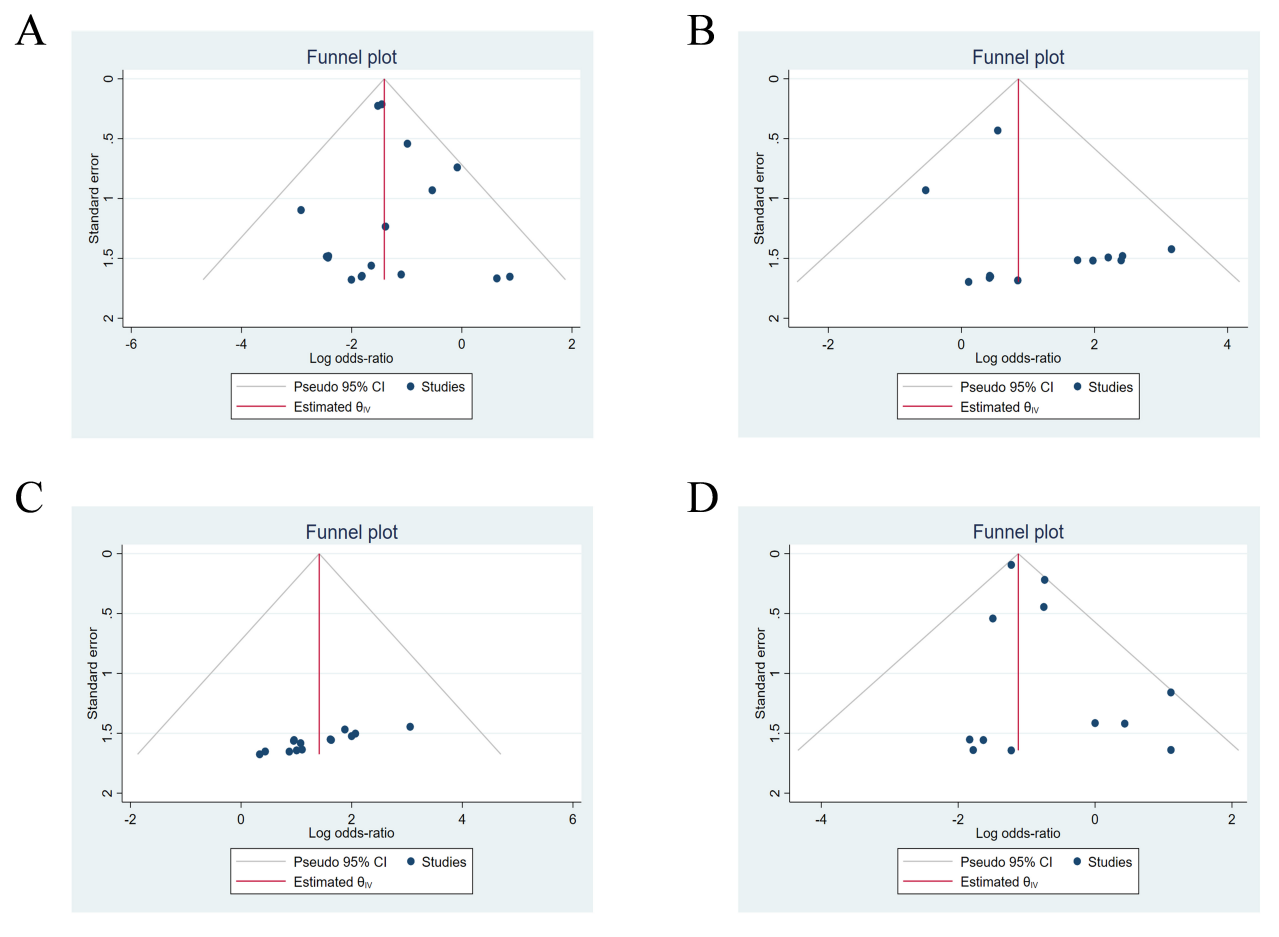
**Funnel plots for publication bias across different complications**. (A) Puncture site events. (B) Lead/device dislodgment. (C) Pocket-related complications. (D) Pericardial effusion/perforation.

**Fig. 3.**
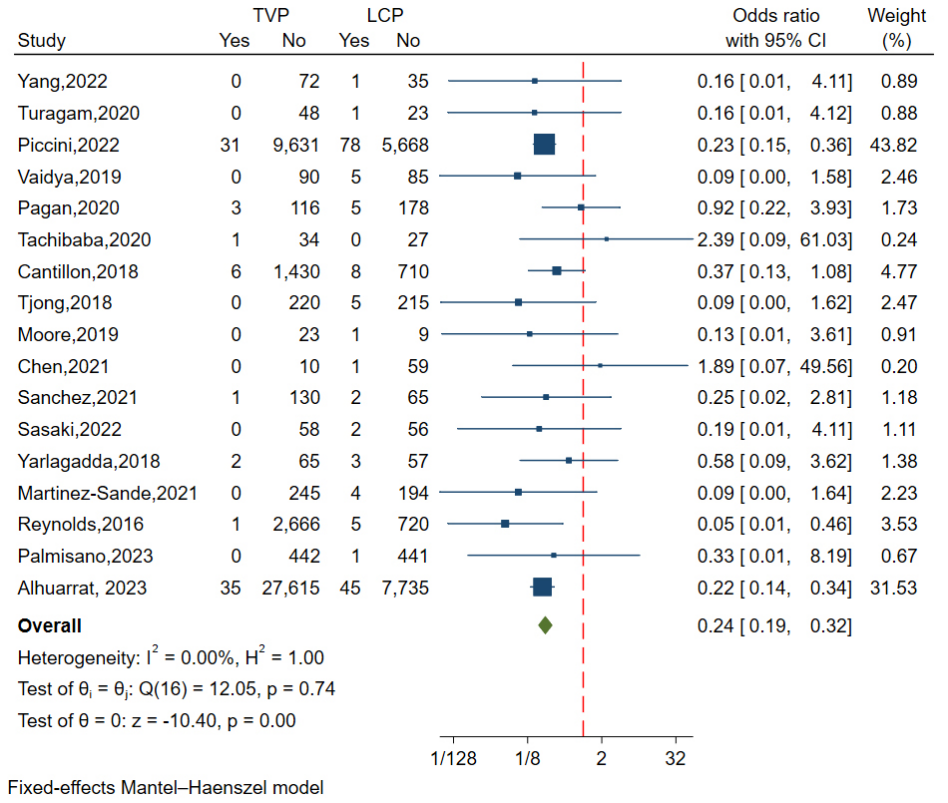
**Forest plot of puncture site event comparisons between LCP and TVP**. This plot presents the odds ratios and confidence intervals, showing lower incidence rates of puncture site events in the TVP group compared to the LCP group. Abbreviations: LCP, leadless cardiac pacemaker; TVP, transvenous pacemaker.

A total of seventeen studies involving 78,938 patients analyzed puncture site events. The I^2^ value, indicating heterogeneity, was 0.00% (*p* = 0.74), which signifies negligible variation between the studies. The analysis of funnel plots and regression-based Egger test revealed no significant evidence of publication bias. (*p* = 0.699) (Fig. 2A). A meta-analysis using a fixed-effects model revealed that the incidence of puncture site events was significantly lower in the TVP group compared to the LCP group (OR = 0.24, 95% CI: 0.19–0.32, *p* < 0.01) (Fig. 3).

#### 3.3.2 Lead/device Dislodgement

**Fig. 4.**
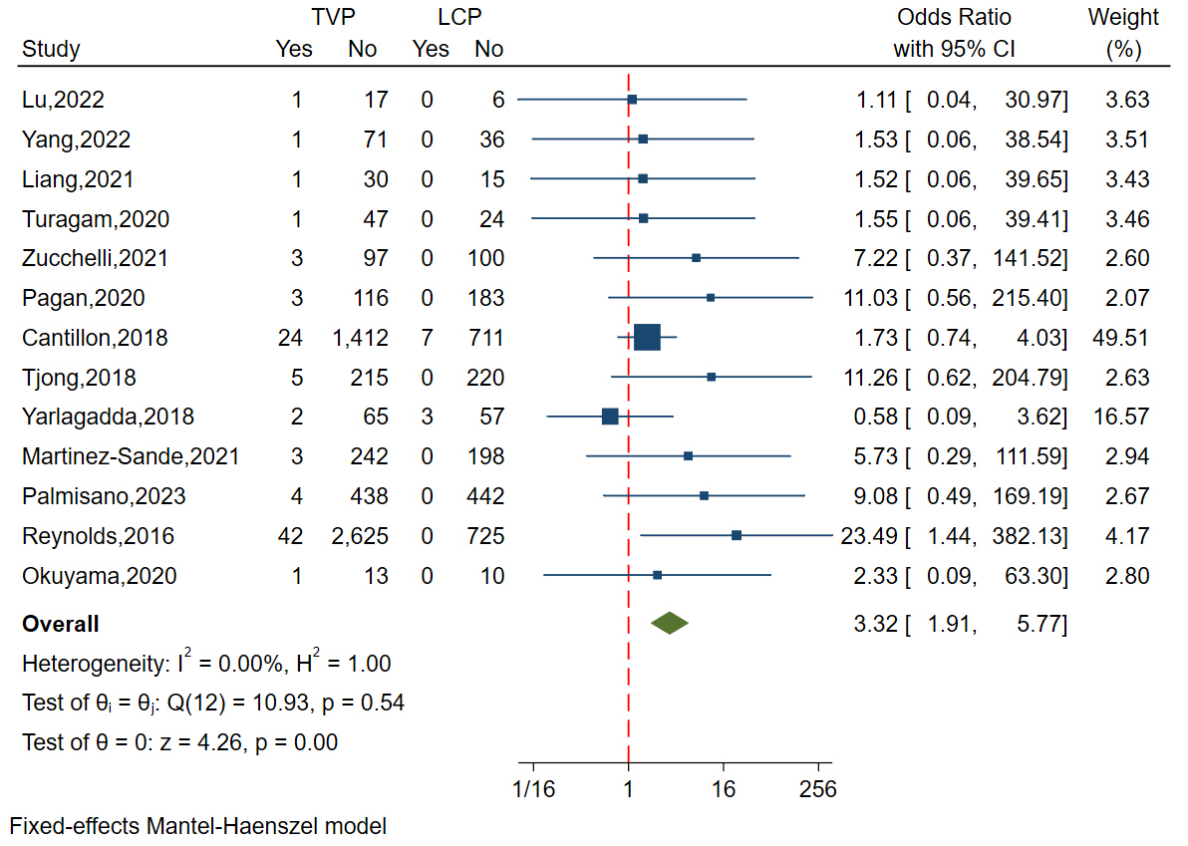
**Forest plot comparing lead or device dislodgement rates between LCP and TVP**. This plot displays the odds ratios and confidence intervals, indicating a significantly lower incidence of dislodgment in the LCP group compared to the TVP group. Abbreviations: LCP, leadless cardiac pacemaker; TVP, transvenous pacemaker.

A total of ten studies, including 6271 patients, analyzed the incidence of lead or device dislodgement. These studies demonstrated minimal heterogeneity (I^2^ = 0.00%, *p* = 0.54), supporting the use of a fixed-effects model. Analysis of funnel plots and regression-based Egger test indicated no publication bias (*p* = 0.162) (Fig. 2B). The results indicated a significantly lower incidence of lead or device dislodgement in the LCP group compared to the TVP group (OR = 3.32, 95% CI: 1.91–5.77, *p* < 0.01) (Fig. 4).

#### 3.3.3 Infective Endocarditis

**Fig. 5.**
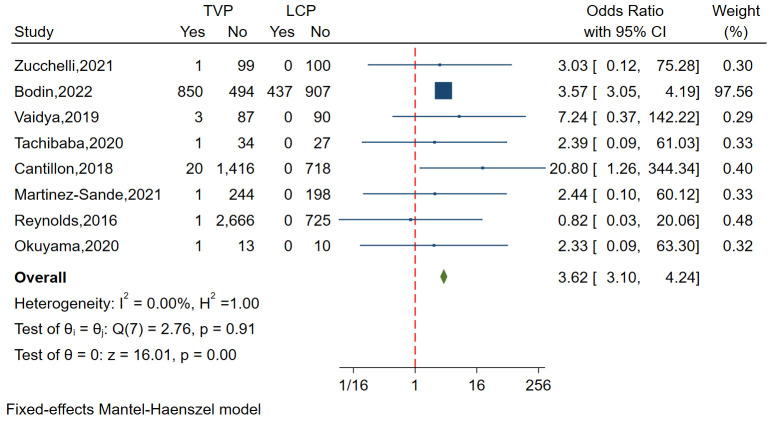
**Forest plot comparing the incidence of infective endocarditis between LCP and TVP**. This plot visualizes the odds ratios and confidence intervals, highlighting a significantly lower rate of infective endocarditis in the LCP group. Abbreviations: LCP, leadless cardiac pacemaker; TVP, transvenous pacemaker.

A total of Eight studies involving 8404 patients analyzed the incidence of infective endocarditis, demonstrating minimal heterogeneity (I^2^ = 0.00%, *p* = 0.91). Funnel plots and regression-based Egger test indicated no publication bias (*p* = 0.984). The analysis showed a significantly lower incidence of infective endocarditis in the LCP group compared to the TVP group (OR = 3.62, 95% CI: 3.10–4.24, *p* < 0.01) (Fig. 5).

#### 3.3.4 TR or Dysfunction

**Fig. 6.**
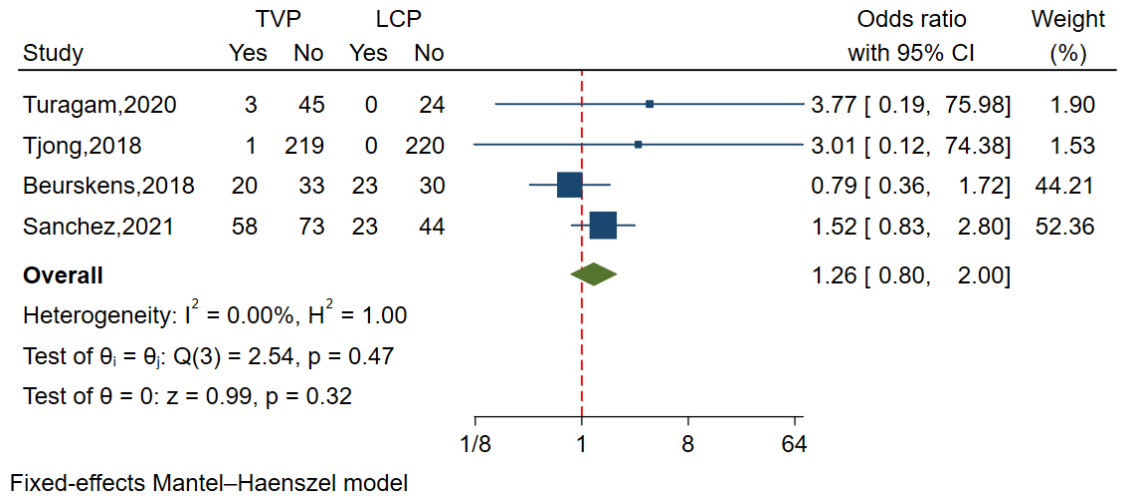
**Forest plot comparing the incidence of TR or dysfunction between LCP and TVP**. This plot visualizes the odds ratios and confidence intervals, showing a marginally lower incidence in the LCP group. Abbreviations: LCP, leadless cardiac pacemaker; TVP, transvenous pacemaker; TR, tricuspid regurgitation.

A total of four studies comprising 816 patients analyzed the incidence of TR or dysfunction, revealing minimal heterogeneity (I^2^ = 0.00%, *p* = 0.470). Funnel plots and a regression-based Egger test indicated no publication bias (*p* = 0.481). The results indicated that the incidence of TR or dysfunction was slightly lower in the LCP group compared to the TVP group, although this difference was not statistically significant (OR = 1.26, 95% CI: 0.80–2.00, *p* = 0.32) (Fig. 6).

#### 3.3.5 Infection

**Fig. 7.**
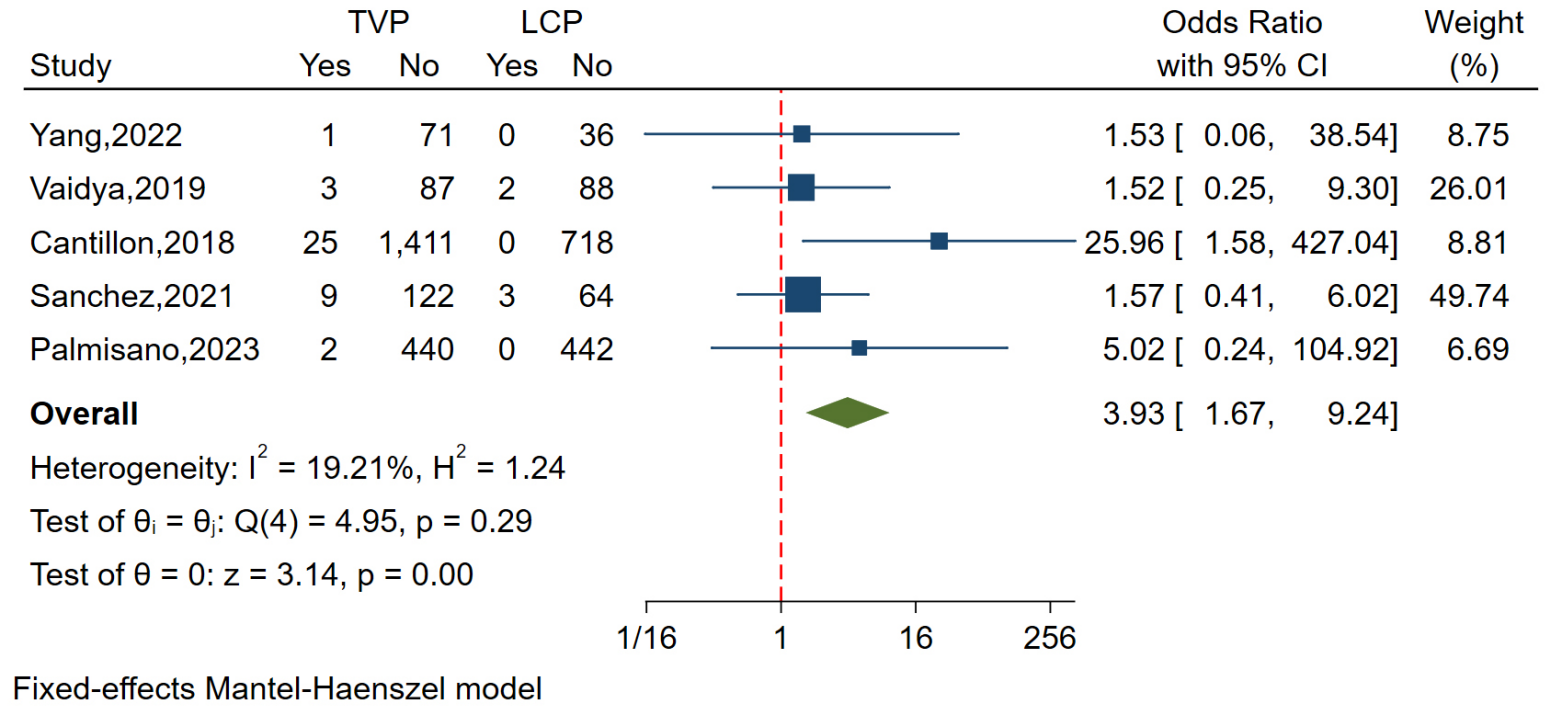
**Forest plot comparing infection incidences between LCP and TVP**. This plot visualizes the odds ratios and confidence intervals, indicating a significantly lower incidence of infection in the LCP group. Abbreviations: LCP, leadless cardiac pacemaker; TVP, transvenous pacemaker.

A total of five studies involving 3082 patients analyzed the incidence of infection and showed relatively small heterogeneity (I^2^ = 19.21%, *p* = 0.290). Funnel plots and a regression-based Egger test showed no publication bias (*p* = 0.249). The analysis revealed a significantly lower incidence of infection in the LCP group compared to the TVP group (OR = 3.93, 95% CI: 1.67–9.24, *p* < 0.01) (Fig. 7).

#### 3.3.6 Increased Right Ventricle Pacing Threshold

**Fig. 8.**
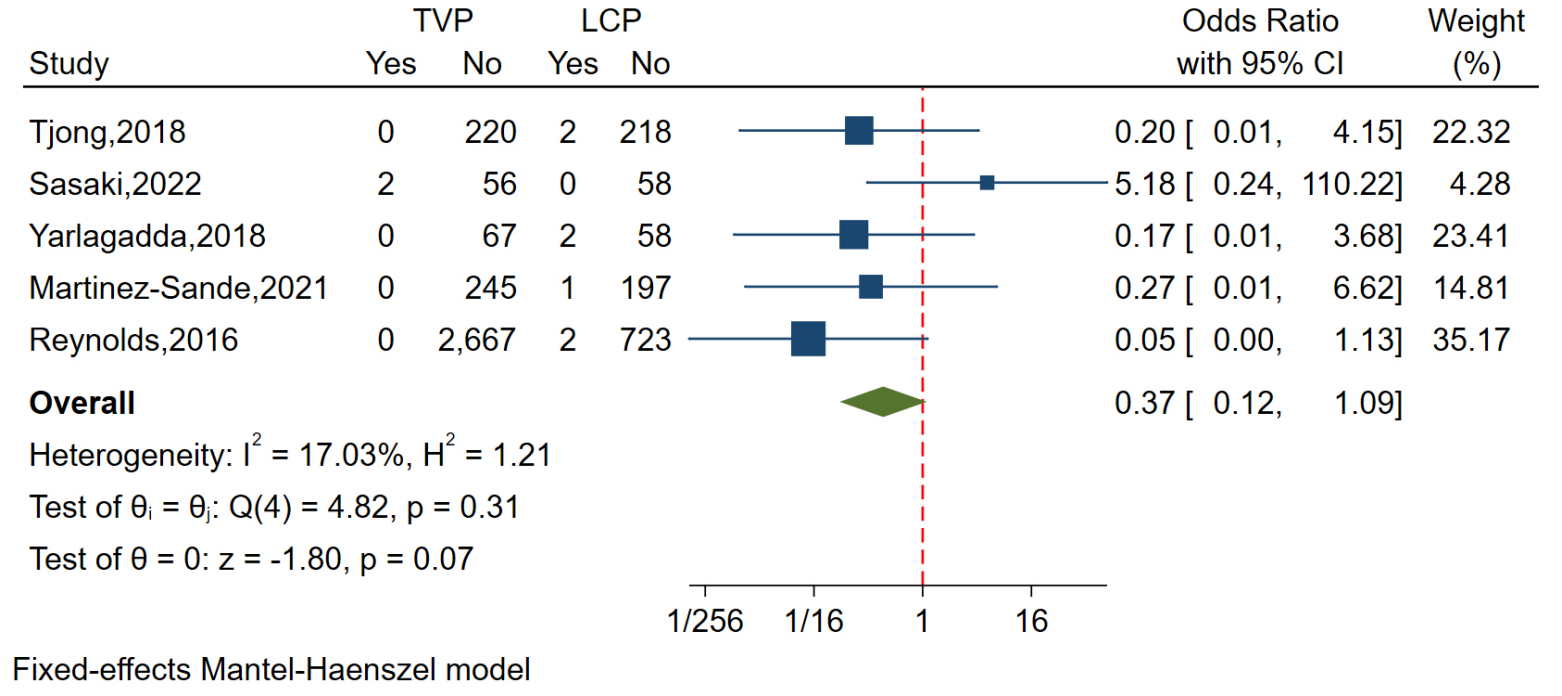
**Forest plot comparing RV pacing threshold increases between LCP and TVP**. This plot visualizes the odds ratios and confidence intervals, indicating a marginally higher incidence of increased RV pacing thresholds in patients with LCP. Abbreviations: LCP, leadless cardiac pacemaker; TVP, transvenous pacemaker; RV, right ventricle.

A total of five studies involving 4518 patients analyzed the incidence of increased RV pacing threshold, demonstrating relatively small heterogeneity (I^2^ = 17.03%, *p* = 0.310). Funnel plots and regression-based Egger test revealed no evidence of publication bias (*p* = 0.896). Overall, the study found a slightly higher rate of increased RV pacing threshold in patients with LCP compared to those with TVP, although this difference was not statistically significant (OR = 0.37, 95% CI: 0.12–1.09, *p* = 0.07) (Fig. 8).

#### 3.3.7 Pericardial Effusion or Perforation

**Fig. 9.**
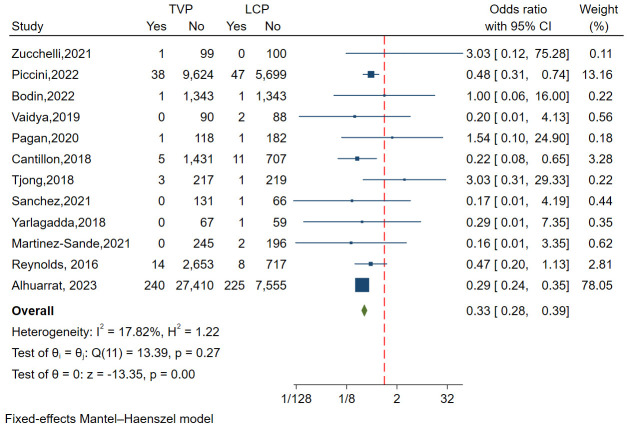
**Forest plot comparing of pericardial effusion or perforation between LCP and TVP**. This plot visualizes the odds ratios and confidence intervals, highlighting a significantly higher incidence of pericardial complications in the LCP group. Abbreviations: LCP, leadless cardiac pacemaker; TVP, transvenous pacemaker.

A total of twelve studies, with 53,243 patients, assessed the incidence of pericardial effusion or perforation, demonstrating relatively small heterogeneity (I^2^ = 17.82%). Funnel plots and regression-based Egger test revealed no publication bias (*p* = 0.701)(Fig. 2D). A fixed-effects model showed that the incidence of pericardial effusion/perforation was significantly higher in the LCP group compared to the TVP group (OR = 0.33, 95% CI: 0.28–0.39, *p* < 0.01) (Fig. 9).

#### 3.3.8 Thrombosis and Embolism

**Fig. 10.**
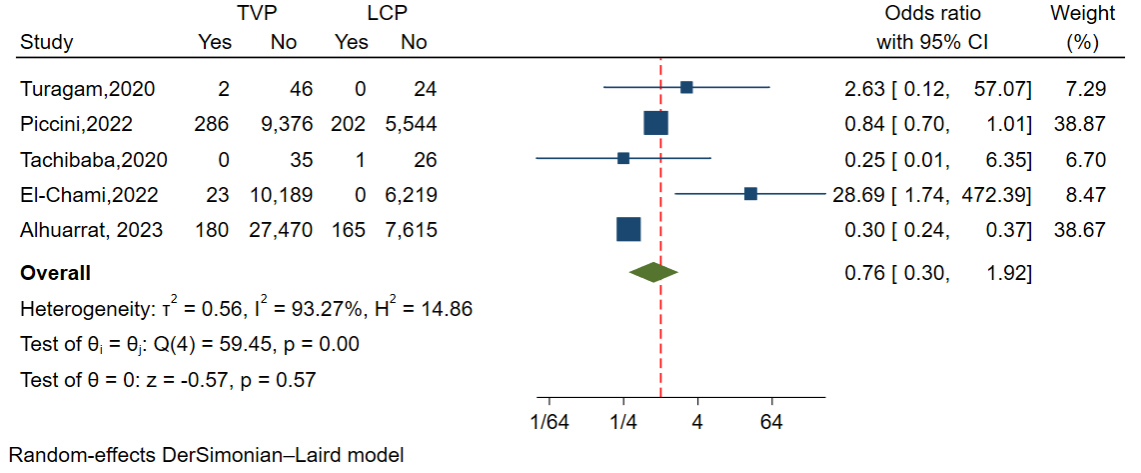
**Forest plot comparing incidences of thrombosis and embolism between LCP and TVP**. This plot visualizes the odds ratios and confidence intervals, indicating a lower but not statistically significant incidence of thrombosis and embolism in the LCP group. Abbreviations: LCP, leadless cardiac pacemaker; TVP, transvenous pacemaker.

A total of five studies with 61,184 patients evaluated the incidence of thrombosis and embolism, exhibiting relatively large heterogeneity (I^2^ = 93.25%, *p* <0.01). Funnel plots and regression-based Egger test revealed no publication bias (*p* = 0.136). The random-effect model revealed the incidence of thrombosis and embolism was lower in the LCP group than in the TVP group, although this difference was not statistically significant (OR = 0.76, 95% CI: 0.30–1.92, *p* = 0.57) (Fig. 10).

#### 3.3.9 Pocket-related Complications

**Fig. 11.**
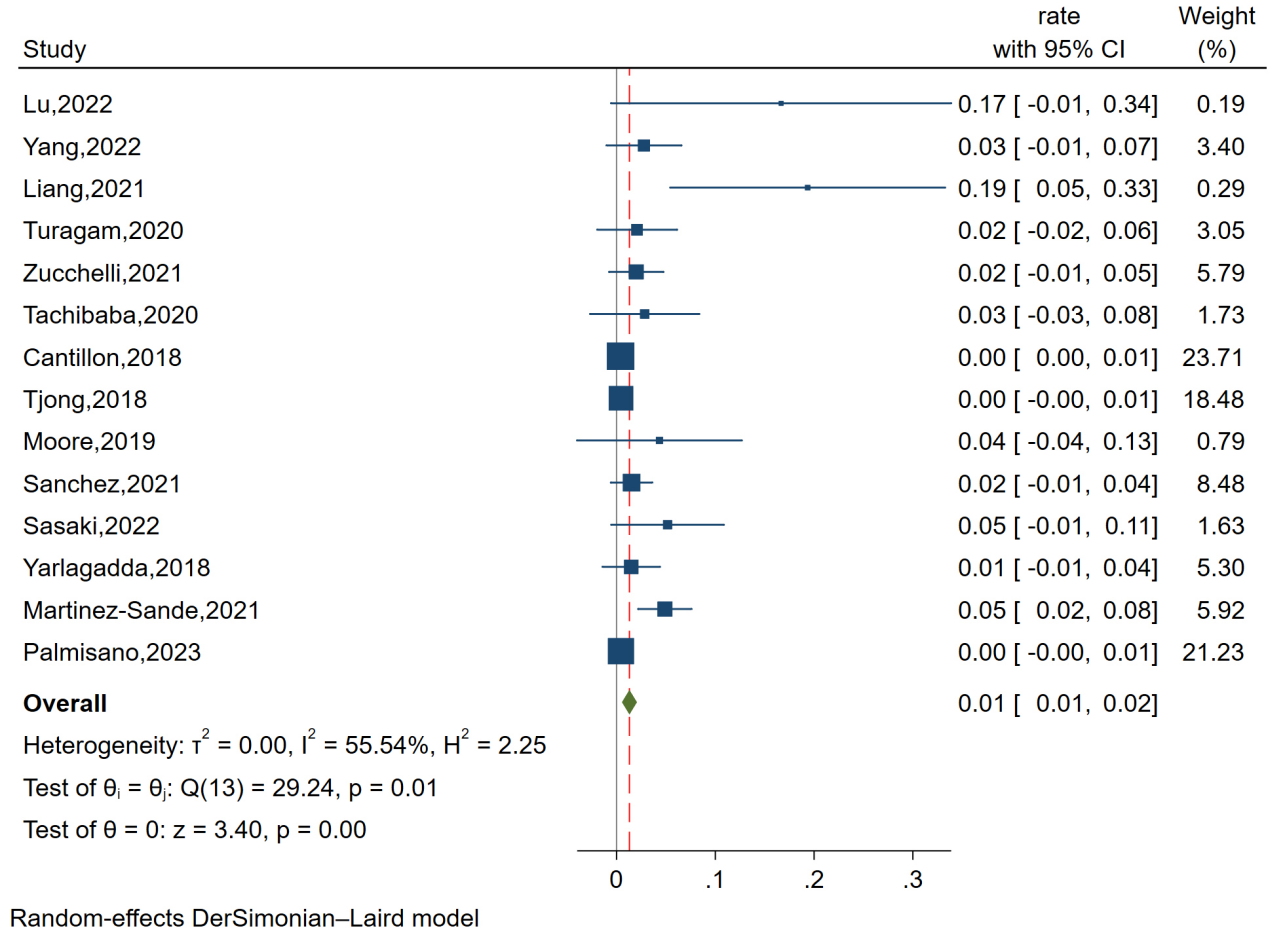
**Forest plot showing the incidence of pocket-related complications in the TVP group**. This plot visualizes the rate of pocket-related complications, highlighting the consistent occurrence within the TVP group. Abbreviations: TVP, transvenous pacemaker.

A total of fourteen studies with 2926 patients evaluated the incidence of pocket-related complications via pooled-analysis. The analysis of funnel plots and regression-based Egger test revealed no significant evidence of publication bias. (*p* = 0.144) (Fig. 2C). The studies had I^2^ value of 55.54%, indicating relatively large heterogeneity, warranting a random-effects model. The forest plot indicated the incidence of pocket-related complications after TVP was 1% (OR = 0.01, 95% CI: 1%–2%, *p* < 0.01) (Fig. 11).

## 4. Discussion

This meta-analysis consolidates and expands upon existing evidence regarding the comparative safety profiles of LCPs and TVPs across a range of clinical endpoints. Our findings affirm the nuanced nature of pacemaker selection, highlighting a differential risk pattern associated with each device type. These results underscore the importance of considering individual patient conditions and clinical scenarios when choosing the most appropriate pacemaker technology.

A total of 24 observational studies were included in this review, demonstrating no publication bias across all endpoints. The heterogeneity among the studies was relatively low for pericardial effusion or perforation, puncture site events, infective endocarditis, lead or device dislodgment, TR or dysfunction, any infection, and increased RV pacing threshold. However, the heterogeneity was relatively high for thrombosis and embolism, and pocket-related complications. Notably, the incidences of infective endocarditis, lead or device dislodgment, any infection, and pocket-related complications were lower in the LCP group than in the TVP, while the incidences of puncture site events and pericardial effusion or perforation was lower in the TVP group than in the LCP group.

Recent findings by Shtembari *et al*. [9] suggest that LCPs are associated with a lower incidence of re-intervention, device dislodgment, pneumothoraxes, and overall complications supporting their safety compared to TVPs. However, their analysis also indicates higher rates pericardial effusion in the LCP group. This review extends and diverges from these findings in several crucial aspects. Our analysis includes 17 articles, retrieved up to April 12, 2022, with 14 directly comparing the complications between the two pacemaker types. Additionally, our review incorporates a broader international perspective, analyzing 20 studies published in English and four in Chinese. Importantly, this review expands the scope of examined clinical endpoints to include TR or dysfunction, pocket-related complications, and any infection, thereby providing a more comprehensive assessment of the comparative risks associated with each pacemaker type.

In this study, LCP implantation was associated with a lower risk of several complications, including infective endocarditis, lead or device dislodgment, any infection, and pocket-related complications, corroborating previous findings. Notably, the absence of a lead and subclavian pocket in LCP eliminates their related complications [7, 8]. Previous studies have shown that the incidence of pocket-related complications related to TVP range between 2.90%–4.50% [21, 25, 34]. However, our pooled analysis showed a lower incidence rate of 1% (95% CI: 1%–2%), which may be attributed to the limited number of studies included in this review. Additionally, the simpler surgical procedure associated with LCPs, involving fewer steps and less trauma, contributes to their safety profile [8]. Previous studies have also suggested that the absence of intrinsic wires in LCPs can reduce the risk of infectious endocarditis [20], further supporting the role of LCPs in preventing infections and endocarditis [35].

Although LCPs reduce lead and pocket complications, they increase the risk of pericardial effusion/perforation (P/E) cardiac injury, ranging from 0.9% to 1.6% as reported in previous studies [7, 32, 36]. Furthermore, P/E can lead to severe consequences. For instance, among 563 P/E cases who underwent implantation (extracted from FDA’s Manufacturer and User Facility Device Experience [MAUDE] database between 2016 and July 2021), 150 patients died (27%), 499 patients suffered cardiac tamponades (89%), 64 patients had pericardial effusions (11%), and 146 patients (26%) required emergency surgery within 30 days [37]. Additionally, half of the perforations were associated with device and operator errors.

Device positioning is a critical factor influencing the incidence of P/E, often cited as the second most common device-related issue [38, 39, 40]. The use of an apical site was associated with a higher incidence of complications when compared to a septal location (52% vs 33%) in the post-approval registry [41]. The standard positioning method involves implanting the device at the center of the cardiac silhouette in the right anterior oblique (RAO) view and towards the left in the left anterior oblique (LAO) view. However, challenges persist; for instance, 17.6% of devices directed at the free wall in the left lateral view did not adhere to the optimal placement strategies [42]. Hai J *et al*. [41] attempted to refine this by directing the device away from the sternum in the left lateral view. However, the implant sites were not confirmed via computed tomography (CT) or echocardiogram, leading to one reported perforation (2%) [41]. As a result, Li *et al*. [42] suggested that routine right ventriculography should be performed to improve the accuracy of leadless pacemaker implantation in the RV midseptum. Despite these measures, approximately 6.2% of devices were still not correctly positioned in the mid-septum after guiding via right ventriculography [42]. The difficulty of mid-septum positioning could be attributed to inherent cardiac rotation. This highlights the necessity for enhanced implantation skills to improve mid-septum positioning and thus reduce the risks of perforation and cardiac effusion.

Both endocardial pacemaker leads and RV pacing are recognized as potential contributors to dysfunction of the tricuspid and mitral valves, as well as overall cardiac dysfunction [17]. Previous study has shown that TVP implantation may lead to or exacerbate TR in approximately 10%–45% of cases, possibly due to damage to tricuspid leaflets or sub-valvular apparatus during lead implantation and long-term mechanical stress exerted by the lead across valve [2]. Although LCP avoids the impact of traditional pacemaker leads on tricuspid valve mechanics, it does not entirely eliminate the risk of tricuspid valve dysfunction, which can be a chronic process potentially influenced by different follow-up durations [8, 10, 11, 12, 13, 14, 15, 16, 17, 18, 20, 21, 22, 23, 24, 25, 26, 27, 28, 29, 30, 31, 32, 33]. In our study, however, the incidence of TR or dysfunction was found to be slightly lower in the LCP group compared to the TVP group.

With advancements such as the Micra AV and atrial LCP, the scope of LCP implantation has expanded beyond patients who traditionally required only single-chamber pacing. As a relatively new technology, LCP implantation comes with a learning curve. The proficiency of the operator plays a crucial role in the incidence of post-operative complications, such as pericardial effusion or perforation [18]. It is expected that as implantation skills improve with experience, procedure-related complications will decrease. In conclusion, LCP represents a promising alternative to TVP, particularly as operator the implantation skills improve over time.

## 5. Limitations

This study has some limitations. Few clinical application of leadless pacemakers is in its early stages, resulting in few prospective studies due to high research costs and complex ethical, regulatory, and patient preference issues. Consequently, most available data derive from observational, often retrospective studies, which are subject to various biases. Additionally, variations in devices among patients can further confound results. To overcome these limitations, RCTs are necessary, although such trials for these devices are costly compared to traditional pacemakers. Moreover, leadless pacemakers have a slightly elevated risk of pericardial effusion (sometimes requiring surgical intervention), and the potential for pacemaker-induced cardiomyopathy, particularly with the transition to conduction system pacing. These risks might initially seem to outweigh the benefits of leadless pacing. Nevertheless, given the millions of devices implanted annually, the expectation of randomized controlled trials is not unreasonable. While single-chamber leadless pacemakers may seem dispensable, the benefits of dual-chamber versions become evident in RCTs. Ongoing technological advancements and further research are expected to improve the efficacy and safety of leadless pacemakers.

## 6. Conclusions

Compared with TVP, LCP implantation has a lower risk of infective endocarditis, lead or device dislodgment, any infection, and pocket-related complications, but it presents a higher risk of puncture site complications and pericardial effusion or perforation. Therefore, this study contributes to the development of personalized therapy strategies tailored to specific patient conditions. However, to gain more definitive insights, further research in the form of large-scale, multi-central RCTs with long-term follow-up is essential.

## Data Availability

In the review, the template data collection forms, data extracted from the included studies, the data used for all analyses, as well as the analytical code are available to all.

## Abbreviations

LCP, leadless cardiac pacemaker; TVP, transvenous pacemaker; OR, odds ratio; CI, confidence interval; RV, right ventricle; DVT, deep venous thrombosis; AV, arterial-venous; RCT, randomized controlled trials; OSs, observational studies; NOS, Newcastle-Ottawa quality assessment scale; MD, mean difference; TR, tricuspid regurgitation; FDA, Food and Drug Administration.

## Supplementary Material

Supplementary material associated with this article can be found, in the online version, at https://doi.org/10.31083/j.rcm2510359.
