# Supplementary material for: Comparison of Postoperative Outcomes between Leadless and Conventional Transvenous Pacemakers Implantation: An Up-to-Date Meta-analysis
Source: Rev Cardiovasc Med. 2024 Oct 9;25(10):359. doi: 10.31083/j.rcm2510359 (PMC11522773; doi:10.31083/j.rcm2510359)
Supplement: Supplementary file 1 [file 2153-8174-25-10-359-s1.zip › 2153-8174-25-10-359-s1/Supplementary material.docx]

**Supplementary Table 1.** Literature searching strategy.

| Database | Searching strategy |
| --- | --- |
| PubMed | (((((leadless pacemaker) OR (leadless pacing)) OR (micra)) OR (nanostim)) OR (transvenous pacemaker)) OR (traditional pacemaker) |
| Embase | ('leadless pacemaker':ab,ti OR 'leadless pacing':ab,ti OR micra:ab,ti OR nanostim:ab,ti) AND 'transvenous pacemaker':ab,ti OR 'traditional pacemaker':ab,ti |
| Ovid Medline | Leadless pacemaker.m_titl. or leadless pacing .m_titl. or micra .m_titl. and transvenous pacemaker.m_titl.or traditional pacemaker .m_titl. |
| Cochrane Database | (leadless pacemaker):ti,ab,kw OR (leadless pacing):ti,ab,kw OR (micra):ti,ab,kw OR (nanostim):ti,ab,kw AND (traditional transvenous pacemaker):ti,ab,kw |
| Web of science | (((((TS=(leadless pacemaker)) OR TS=(leadless pacing)) OR TS=(micra)) OR TS=(nanostim)) AND TS=(transvenous pacemaker)) OR TS=(traditional pacemaker) |
| CNKI | (篇名=无导线起搏器) OR (篇名=micra) OR (篇名=nanostim) AND (篇名=传统经静脉起搏器) |
| Weipu | (M=(无导线起搏器 OR micra OR nanostim))AND M=(传统起搏器 OR 经静脉起搏器) |
| Wanfang | 主题:("无导线起搏器") or 题名或关键词:("micra") or 题名或关键词:("nanostim") or 题名或关键词:("传统经静脉起搏器") |
